# Refinement of Animal Model of Colorectal Carcinogenesis through the Definition of Novel Humane Endpoints

**DOI:** 10.3390/ani11040985

**Published:** 2021-04-01

**Authors:** Rita Silva-Reis, Ana I. Faustino-Rocha, Mariana Gonçalves, Catarina Castro Ribeiro, Tiago Ferreira, Carla Ribeiro-Silva, Lio Gonçalves, Luís Antunes, Carlos Venâncio, Rita Ferreira, Adelina Gama, Paula A. Oliveira

**Affiliations:** 1 Center for the Research and Technology of Agro-Environmental and Biological Sciences (CITAB), University of Trás-os-Montes and Alto Douro (UTAD), 5000-801 Vila Real, Portugal; ritareis96@hotmail.com (R.S.-R.); anafaustino.faustino@sapo.pt (A.I.F.-R.); ma1ri2a3na4@gmail.com (M.G.); catarinaribeiro8f@gmail.com (C.C.R.); tiagoterras55@gmail.com (T.F.); carla_ribeiro_silva@hotmail.com (C.R.-S.); lantunes@utad.pt (L.A.); cvenanci@utad.pt (C.V.); 2 Department of Zootechnics, School of Sciences and Technology, University of Évora, 7000-812 Évora, Portugal; 3 Engineering Department, University of Trás-os-Montes and Alto Douro (UTAD), 5000-801 Vila Real, Portugal; lgoncalv@utad.pt; 4 Institute for Systems and Computer Engineering, Technology and Science, 4200-465 Porto, Portugal; 5 Department of Veterinary Sciences, University of Trás-os-Montes and Alto Douro (UTAD), 5000-801 Vila Real, Portugal; agama@utad.pt; 6 Department of Animal Sciences, University of Trás-os-Montes and Alto Douro (UTAD), 5000-801 Vila Real, Portugal; 7 Associated Laboratory for Green Chemistry (REQUIMTE), Department of Chemistry, University of Aveiro (UA), 3810-193 Aveiro, Portugal; ritaferreira@ua.pt; 8 Animal and Veterinary Research Center (CECAV), University of Trás-os-Montes and Alto Douro (UTAD), 5000-801 Vila Real, Portugal

**Keywords:** colorectal cancer, rat, welfare, 1,2-dimethylhydrazine

## Abstract

**Simple Summary:**

Ensuring animal welfare is essential in protocols using laboratory animals. Applying a score sheet with 14 biological parameters, we assessed the welfare of 29 male Wistar rats used as models of colorectal carcinogenesis (CRC). We found a uniformity of characteristics preceding the premature animals’ death, including an increase of 10% in body weight, swollen abdomen, diarrhea, and priapism. In addition, we observed that surface abdominal temperature was higher in animals with CRC. We considered that the parameters already described in other cancer models are insufficient and considered assessing the abdominal temperature, priapism, and sudden increase in the body weight in the model of CRC.

**Abstract:**

This study aimed to define appropriate humane endpoints (HEs) for an animal model of colorectal carcinogenesis (CRC). Twenty-nine male Wistar rats were divided into two control groups (CTRL1 and CTRL2) injected with ethylenediamine tetraacetic acid (EDTA)–saline solutions and two induced groups (CRC1 and CRC2) injected with 1,2-dimethylhydrazine (DMH) for seven weeks. A score sheet with 14 biological parameters was used to assess animal welfare. Groups CRC1 and CTRL1 and groups CRC2 and CTRL2 were euthanized 11 and 17 weeks after the first DMH administration, respectively. Five animals from the induced groups died unexpectedly during the protocol (survival rates of 75.0% and 66.7% for groups CRC1 and CRC2, respectively). The final mean body weight (BW) was smaller in the CRC groups when compared with that in the CTRL groups. A uniformity of characteristics preceding the premature animals’ death was observed, namely an increase of 10% in mean BW, swollen abdomen, diarrhea, and priapism. The surface abdominal temperature of group CRC2 was significantly higher, when compared with that of group CTRL2. The parameters already described in other cancer models proved to be insufficient. For the CRC model, we considered assessing the abdominal temperature, priapism, and sudden increase in the BW.

## 1. Introduction

Several species can be used for in vivo studies. However, mice and rats are the most used due to their numerous advantages as following: their biochemistry, physiology, and genetic anatomy are well studied; their easy manipulation, low cost, and small size make them easy to maintain in experimental conditions; and the results are easily transposed to humans [1,2,3]. The rat, as an animal model, confers some advantages over the mouse as follows: its size facilitates the monitoring of physiological parameters, and the rat has a larger memory and learns faster than the mouse. These can be important factors according to the objectives of a previous study [4]. Experiments with laboratory rodents raise many ethical questions. Several studies cause pain and stress, and it is important to minimize them because these factors directly influence experimental results [5]. In order to improve animal welfare in experimental trials, in 1959, Russel and Burch introduced the concept of the 3Rs (i.e., reduce, refinement, and replacement), suggesting that studies using animals should only be performed after testing all possible alternative methods, the number of animals should be reduced to a minimum and the procedures should be refined, being as strict as possible [6]. Although animal studies continue to be carried out, public concern about the welfare and scientific validation of research on laboratory animals has been growing. Thus, it becomes essential to define humane endpoints that purpose to implement the 3Rs [7,8]. Humane endpoints (HEs) aim to establish critical limits that indicate the moment when the animal reaches a certain level of suffering and must be removed from the protocol [9]. Early HEs should ideally be centered on defined scientific information in addition to welfare-centered criteria [7]. Indeed, the Canadian Council on Animal Care (CCAC) established a list of guidelines aiming to assess the animals’ welfare through appropriate endpoints in the experiment, assigning a score to each parameter evaluated for research, teaching and testing procedures with animals. Specific guidelines for animal models of stress, toxicological studies, infectious diseases, and cancer models were also proposed [10,11,12]. Later, HEs for experimental assays on cancer were established by Oliveira et al. [5] and Faustino-Rocha et al. [9].

In order to address all ethical and animal welfare issues in an animal model of chemically induced colorectal carcinogenesis (CRC), this study aimed to define a clear HE scoring system to identify signs of animal suffering before the onset of severe effects of the disease. We hypothesize that HE scoring systems previously proposed for animal models of cancer [5,9] can be applied for chemically induced CRC after the integration of specific parameters, such as abdominal temperature, priapism, and body weight (BW) gain.

## 2. Materials and Methods

### 2.1. Animals and Chemicals

Twenty-nine male Wistar rats (*Rattus norvegicus*) with seven weeks of age and weighing 200–250 g were obtained from Charles River (France). Dimethylhydrazine (DMH) was obtained from Sigma-Aldrich (St. Louis, MO, USA) and prepared immediately before its use in a 1 mM ethylenediamine tetraacetic acid (EDTA)–saline solution as a vehicle [13]. The animals were housed in polycarbonate cages (3–5 animals per cage) and properly identified, with smooth surfaces and rounded corners (1500U Eurostandard Type IV S, Tecniplast, Buguggiate, Italy). Corncob was used for bedding, and it was changed weekly. Polyvinyl chloride tubes were used to enrich the animals’ environment. The animals were kept under controlled conditions of temperature (20 ± 2 °C) and relative humidity (50 ± 10%) with a 12 h light/12 h dark cycle.

### 2.2. Ethics Statement

All ethic issues were reviewed by an Ethics Review Body (“*ORBEA—Órgão Responsável pelo Bem-Estar e Ética Animal*” under reference 142-e-CITAB-2017/2017-09-25). This work was carried out in compliance with the Portuguese legislation (Decree-Law No. 113/2013) on the protection of animals used for experimental purposes and the guidelines of the Portuguese Competent Authority (“*DGAV—Direção Geral de Alimentação e Veterinária*”, approval no. 010535).

### 2.3. Experimental Protocol

**Figure 1:**
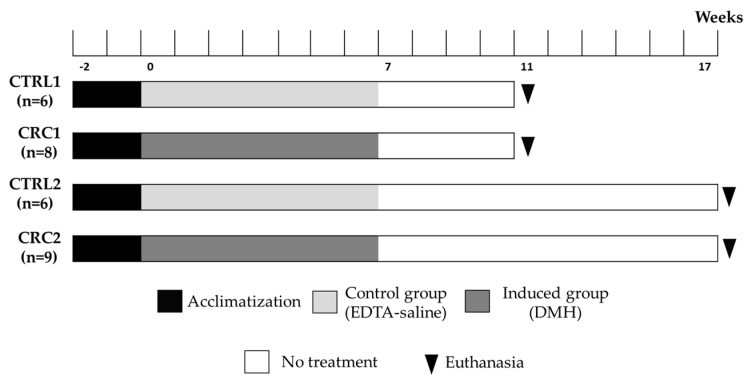
Experimental protocol.

After two weeks of acclimatization, the rats were randomly assigned to four groups. The experimental design was done without “nuisances”, with no need of randomized block designs. The animals had identical characteristics, so the distribution among the four groups was random. Seventeen rats belonging to CRC groups received intraperitoneal injections of DMH (dissolved in a saline solution containing 0.9% NaCl and 1 mM of EDTA as a vehicle; BW: 40 mg/Kg) once a week, during seven consecutive weeks and were named CRC animals. Control groups were intraperitoneally injected with a 1 mM EDTA–saline solution (NaCl 0.9%; B. Braun, Germany) and were named CTRL animals (Figure 1). This protocol was adapted from Zhu et al. [13]. The administrations were performed by a veterinarian and, with a sterile material, a cut of about 2–3 mm was made in the needle cap, allowing the needle to penetrate only the desired length. A higher number of animals were proposed for the induced group due to the exposition of these animals to the carcinogen and considering the higher risk of the death of these animals. All animals had ad libitum access to boiled tap water through capped bottles and standard laboratory diet (Diet Standard 4RF21^®^, Mucedola, Italy) throughout the experiment. The animals were individually weighed; food and water consumptions were also determined using a top-loading scale (Mettler PM4000, LabWrench, Midland, ON, Canada). The mean food consumption for each animal in the cage was measured as the difference between the weight of the food repository at the beginning of the week and that at the end of the week divided by the number of animals in the cage times the number of days. The mean water consumption for each animal was calculated as the difference between the weight of the water bottle at the beginning of the week and that at the end of the week divided by the number of animals in the cage times the number of days [5].

### 2.4. Body Temperature

The body temperature was measured weekly by thermography using a far-infrared camera from FLIR^®^ E8 model E6390 (Tallinn, Estonia). Before the readings, the thermal camera was turned on for 30 min to warm up the camera sensor unit. The animal emissivity was set to 0.98. For each animal, the temperature was measured on the back and the abdomen, and the last one matched the region of cancer development. Temperature readings were always acquired at the same spot. The measurement on the abdomen began immediately after the observation of animals with abdominal swelling. The back temperature was acquired by moving the animal into a black lined cage. The readings were performed, when the animals stood relaxed. The abdominal surface temperature was measured by pointing a hand-held thermographic camera to a contained animal in a supine position inside a black lined cage. These measurements were always performed by the same researcher, and the values were measured at a constant distance of 20 cm, using a ruler to ensure the distance. To avoid animals’ stress when manipulating them, they were handled regularly before and during the study period. All temperature readings were acquired in approximately one minute to avoid any increasing temperature arising from stress responses. In addition, all animals belonging to the control and induced groups were exposed to the same stress levels.

### 2.5. Score Sheet

Following the CCAC guidelines, an adapted table of HEs was applied (Table 1) [5,9]. Specific parameters considered relevant for the CRC model were added, like stool appearance, dimension of damage caused by injections, infection of induced skin lesions, and macroscopic appearance of induced skin lesions. The following parameters were daily observed and registered once a week: body condition, BW, body posture, hair appearance, grooming, mucous color, eyes, ears and whiskers, mental status, response to external stimuli, hydration status, stool appearance, convulsions, dimension of damage caused by injections, macroscopic appearance of induced skin lesions, and the absence or presence of infection in these potentially caused injuries. Weekly, the grids of the cages were removed, and the animals were evaluated for about 5–10 min according to the indicated parameters. Orthopneic posture was assessed by observing the animals’ positions [14], referring to a leaning posture caused by a feeling of shortness of breath. The grooming was evaluated by the color change of the animals’ hair, and the chromodachryorrhea was evaluated according to Mason et al. [15]. The scores attributed to the “Posture” and “Eyes, Ears, and Whiskers” parameters in Table 1 were intended to estimate animal pain, being the latter based on the Rat Grimace Scale [16]. To assess the response to external stimuli, the animals’ response to hand clapping above the cages was evaluated. The skin pinch test was performed on the animals’ hair to evaluate the hydration status [9]. Throughout the study, feces presence and appearance were monitored when changing the animals’ beds.

To assign a score to the BW parameter, ponderal gain (PG%) was calculated using the following equation [17]:

(1) $$PG\;\left(\%\right)=\frac{Final\;body\;weight-Initial\;body\;weight}{Final\;body\;weight\times 100}.$$

A value from zero to three was assigned to each parameter. We established that the animals were removed from the protocol and sacrificed if they reached a total HE score of four. The critic limit of score was determined according to the CCAC guidelines. The individual clinical score of each animal was obtained through the sum of the scores assigned to each parameter. Despite this, in Table 1, there were other parameters with a score of three that implied euthanasia. To ensure the success of this assessment, three observers participated in the monitoring of the animals’ welfare, and the observations were made independently and only cross-checked at specific timepoints.

### 2.6. Animals’ Sacrifice

The animals from two groups were euthanized 11 weeks after the first DMH or EDTA–saline administration (groups CRC1 (*n* = 9) and CTRL1 (*n* = 6)), and the other animals from the remaining groups were sacrificed 17 weeks after the first administration (groups CRC2 (*n* = 8) and CTRL2 (*n* = 6); Figure 1). The animals were sacrificed in two timepoints in order to identify preneoplastic and neoplastic lesions, allowing a follow-up of the CRC development. All animals were sacrificed by an intraperitoneal overdose administration of sodium pentobarbital (Eutasil, CEVA, Libourne, France), followed by exsanguination via cardiac puncture, as indicated by the guidelines of the Federation for Laboratory Animal Science Associations (FELASA). A complete necropsy was performed in each animal. All organs were collected and weighed. The intestines were individualized and weighed separately. The colon was opened longitudinally, gently rinsed with saline in order to remove residual bowel contents and fixed flat in 10% buffered formalin.

### 2.7. Histopathological Analysis

After fixation, intestines were processed for routine histopathological analysis. Sections of 4-μm paraffin-embedded samples were stained with hematoxylin and eosin (H&E) and observed by light microscopy. Tissue sections were evaluated by a veterinary pathologist in a blind fashion, and lesions were classified according to the criteria proposed by Nolte et al. [18].

### 2.8. Statistical Analysis

All data (animals’ survival, HE score, BW, food and water consumption, and body temperature) were analyzed using GraphPad Prism^®^ software for Windows (version 8.0.1, San Diego, CA, USA). The mean and the S.D. were calculated for each parameter. The differences between groups were determined using an analysis of variance (ANOVA) followed by a post hoc Bonferroni test for multiple comparisons. The means of HE scores were analyzed using a two-way ANOVA to test the effect of each of the four groups (two control groups and two groups under DMH administration) on the dependent variable, and the endpoints were inferred from the “scores”. To better understand the differences between the four groups, a post hoc Tuckey’s test was carried out. Values with *p* of <0.05 were considered statistically significant. During the animals’ necropsy, those from the groups exposed to DMH had a content in the cecum, which was distended. Therefore, the weight of the full intestine was removed from the animals’ final weight. A Kaplan–Meier curve was performed to estimate the period of survival after the carcinogen administration, to compare the survival between control and induced groups and the survival between induced groups sacrificed at week 11 and that sacrificed at week 17. Once some animals exhibited macroscopic changes in the intestinal tissue and distinct amounts of intestinal content, the animals’ BW reported in this work was the total BW (prior to euthanasia) subtracting the intestinal tissue and its content. HE score statistics was performed according to the following equation:

(2) $$HE\;score\;statistics=\frac{Sum\;of\;the\;total\;scores\;from\;each\;animal}{Number\;of\;animals\;in\;the\;group}.$$

## 3. Results

### 3.1. Animals’ Survival

**Figure 2:**
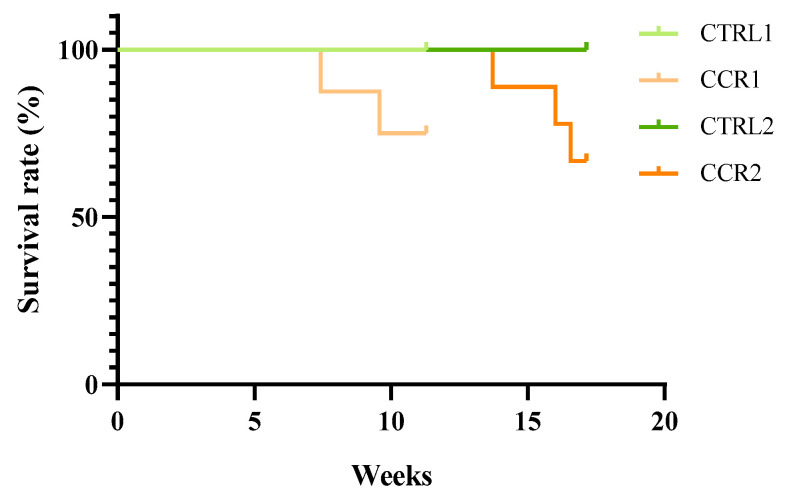
Kaplan–Meier survival curves. Control groups (CTRL1 and CTRL2) injected with ethylenediamine tetraacetic acid (EDTA)–saline and induced groups (CRC1 and CRC2). Groups 1 and 2 were euthanized 11 and 17 weeks after the first administration, respectively.

Five animals from the induced groups died during the protocol: two animals from group CRC1 at weeks 8 and 10 and three animals from group CRC2 at weeks 14, 16, and 17. No major welfare alterations were detected prior to the animals’ death. The animals from both the control groups had a survival rate of 100%, whereas survival rates of 75.0% and 66.7% was observed in groups CRC1 and CRC2, respectively (Figure 2). The Kaplan–Meier survival curves were not significantly different among CRC groups (*p* > 0.05).

### 3.2. HEs Analysis

**Figure 3:**
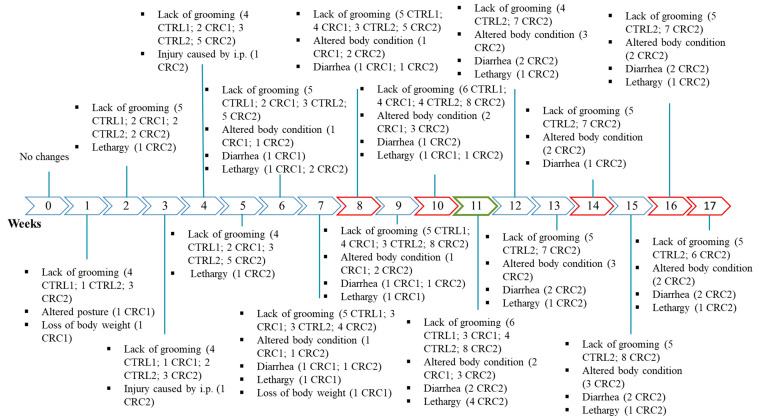
Schedule showing the number of animals with alterations in the humane endpoints (HEs) table. Red color: animal death; green color: 1st euthanasia; R: animals of all groups; control groups (CTRL1 and CTRL2) injected with EDTA–saline solutions and induced groups (CRC1 and CRC2). Groups 1 and 2 were euthanized 11 and 17 weeks after the first administration, respectively. i.p.: intraperitoneal injection. The mean of the scores attributed to biological parameters in all groups can be observed in Table 2. Although no statistic differences were observed among groups (*p* > 0.05), group CTRL1 showed a higher HE score when compared to group CRC1. However, the mean HE score was higher in group CRC2 when compared to that in group CTRL2 (*p* < 0.05).

Six animals reached a score of 3, but none of them reached the critical limit to be humanely sacrificed. Figure 3 presents all altered parameters and the number of animals with those changes. At the beginning of the experiment, before DMH administration, all animals scored 0 for the established HE. The first alterations appeared simultaneously two weeks after the beginning of the experiment in animals from both the control and induced groups, with animals showing the lack of grooming. During the first injection, the lack of grooming was more prevalent in the animals from control groups than in those from the CRC groups. The alterations in the body condition were as following: the most frequent, with some CRC animals showing a swollen abdomen; a BW loss of more than 10%; an alteration in the appearance of the hair, i.e., the lack of grooming; lethargy and the appearance of soft stools and diarrhea. Five animals were also lethargic. Except for the lack of grooming, all alterations were only observed in the animals from the induced groups.

### 3.3. Macroscopic and Histopathological Analysis

At *postmortem* evaluation, no macroscopic lesions were observed in the control groups, whereas several animals from the induced groups exhibited abdominal distension, associated with the segmental distension and impaction of the intestine, affecting small intestine and cecum. No macroscopic evidence of intestinal perforation/rupture or peritonitis was observed. Focal hemorrhagic foci were rarely observed in the distended intestine.

**Figure 4:**
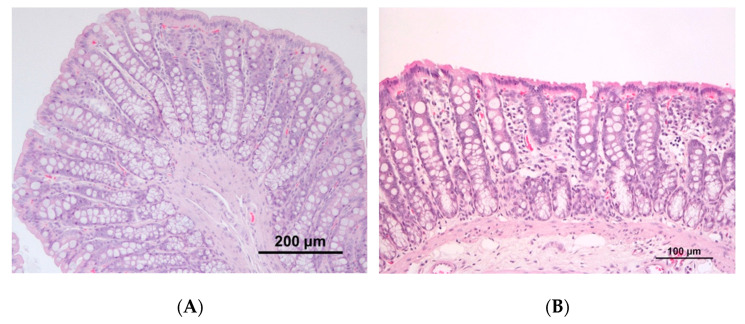

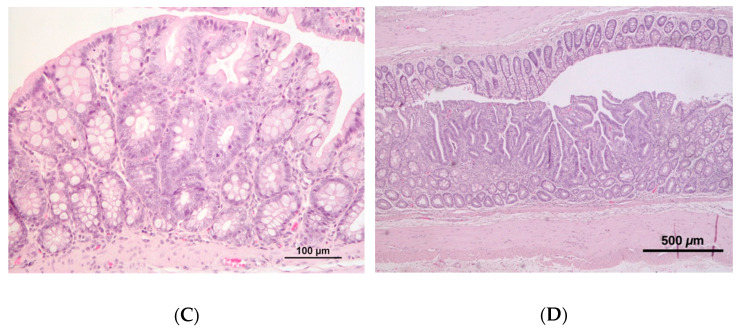
Rat colon histopathology with hematoxylin and eosin (H&E) staining. (**A**) Colon with no alterations in group CTRL1; (**B**) colon showing mild lymphoid inflammatory infiltrate at the mucosa in group CRC1; (**C**) colon with preneoplastic lesions in group CRC2; (**D**) colon with neoplastic epithelial lesion in group CRC2.

The colon of animals from the induced groups presented a mild to moderate mononuclear inflammatory infiltrate with the occasional disruption of architectural structure, while controls were usually characterized by mild inflammatory infiltrate. A moderate to marked mononuclear inflammatory infiltrate was also frequently observed in the small intestine and cecum of animals from both the induced groups. No macroscopic tumors were observed in the colon of rats, either from the control or induced groups; at the histopathological analysis, half of the animals (*n* = 3) belonging to group CRC1 presented preneoplastic lesions, whereas in CRC2 group, both preneoplastic (*n* = 3) and neoplastic (*n* = 1) lesions were observed (Figure 4).

As stated previously, five animals from the induced groups died during the protocol. A postmortem evaluation was possible in four animals, showing lesions of hemorrhagic enteritis. None of these animals presented macroscopic evidence of peritonitis or ascites. Besides the parameters already described, other alterations were registered, such as the increase in animals’ BWs before the appearance of swollen abdomen and priapism. Weekly, the BW increase was detected using Equation (2). All symptoms that preceded animals’ death remained for the week of death of each one. No signs of constipation were observed. Inversely, signs of diarrhea were observed in the anus and the bed. Some of these animals survived several weeks after the first alteration (Table 3). One animal from group CRC1 died at the beginning of the DMH administrations, and it was not possible to observe a pathological pattern or address the cause of death.

In all animals that died precociously, the ponderal weight gain decreased from the acclimatization period where no administrations were performed to the week before the BW increase. The BW of one animal decreased in the week before the BW increase (negative ponderal weight) (Table 4).

### 3.4. Biological Parameters

The final corrected BW of group CTRL2 was higher when compared with that from group CRC1 (*p* < 0.05) (Figure S1, Supplementary Materials). Excepting this difference, no more differences were observed in the initial BW and in the initial and final food and water consumptions among groups (*p* > 0.05). The mean BW variation over time is shown in Figure S1 (Supplementary Materials).

### 3.5. Body Temperature

The mean body temperatures measured by thermography on the animals’ back were similar among groups (*p* > 0.05). However, the mean body temperature measured throughout the experiments by thermography on the animals’ abdomen of group CRC2 was significantly higher than that of group CTRL2 (32.76 ± 0.69 °C and 31.05 ± 1.83 °C, respectively) (*p* < 0.05). No differences were noticed between groups CTRL1 and CRC1 (30.23 ± 2.04 °C and 31.95 ± 1.40 °C, respectively). The mean back and abdominal body temperature variations over time are shown in Figure S2 (Supplementary Materials).

## 4. Discussion

Licensing of research projects with laboratory animals is required by national and international funding entities, as well as by the institutions where works are carried out. The forms filled by the researchers for these purposes included the prediction of the damage and suffering caused to the animals. However, despite the increasing tight demands regarding the use of laboratory animals, the number of scientific publications on the damage caused by certain procedures to laboratory animals is scarce or even nonexistent, making the prediction of damage to be difficult and demonstrating the importance of studies developed for this purpose. This study aimed to propose the appropriate HEs for an animal model of chemically induced CRC. The use of an HE table is fundamental not only to prevent animals’ suffering during the experimental protocol, but also to be considered in the experimental design to prevent the trials being interrupted before the scheduled date.

The high incidence and mortality of CRC combined with the lack of an effective method of early diagnosis and treatment make CRC one of the most relevant cancers to be studied [19]. Chemical carcinogens, such as azoxymethane, DMH, *N*-methyl-*N*-nitro-*N*-nitrosoguanidine, and *N*-methyl-*N*-nitrosourea, are widely used in animal models of cancer, as they mimic the spontaneous development of specific CRC mutations in humans and allow the study of tumor development in long protocols [20,21]. Our study selected the protocol of CRC chemical induction suggested by Zhu et al. [13], since it allows studying the evolution of the disease by including several euthanasia timepoints [13]. However, in the present investigation, DMH administrations were stopped three weeks earlier than in Zhu et al. [13] work, to avoid causing unnecessary suffering and pain to the animals. Compared to Zhu et al. [13], a higher number of animals died precociously in our study, not due to cachexia, but due to hemorrhagic enteritis. These differences may be due to the different ages of the animals, since the animals in the reference work were four weeks old and the animals used in our study were seven weeks old. In fact, a previous work showed that the effects of chemical carcinogens are more severe in older rats [22]. Animal models of cancer are classified as severe [12]. As we intended to early detect pain and suffering, a new HE table was proposed in this work.

In our study, the animals that died suddenly during the study presented hemorrhagic enteritis at the postmortem evaluation. A previous study suggested that diarrhea is a symptom associated with enteritis, as happened in our animals [23]. This can be caused by the presence of *Escherichia coli* in the intestinal mucosa, as it is a pathogen associated with CRC [23].

The use of a score sheet is essential to avoid forgetting the parameters to be evaluated, and it serves to create work habits and encourage their use and registration. During our HE collection, no animal reached the critical limit of four. However, we observed some clinical signs suggestive of disease. Therefore, we assumed that this HE table can be improved to evaluate earlier and with more efficacy the animals’ welfare condition. From these parameters, we observed changes in body condition, hair appearance and grooming, posture, mental status, stool appearance, and dimension of damage caused by injections. Hair appearance and grooming was the parameter showing larger alterations during this study. Self-grooming is a natural behavior, carried out by rodents in an organized and standardized manner, important in maintaining hygiene, thermoregulation and social communication [24]. All the animals showed a decrease in self-grooming after the first intraperitoneal injection; however, the animals from the control groups (CTRL1 and CTRL2) presented a greater lack of grooming, compared to the DMH-exposed groups (CRC1 and CRC2), which contributed to the higher mean HE score in group CTRL1 when compared with that in the group CRC1. To the best of our knowledge, this was not previously reported and requires further investigation. The animals from the control and DMH-exposed groups shared the same manipulation, namely, contention and intraperitoneal administration of the vehicle and housing conditions. There is no easy explanation for such observations. The lack of grooming is commonly related with direct impact from poor animal welfare. However, there are also reports suggesting that rodent self-grooming may be a useful measure of repetitive behavior in some animal models [24]. In such cases, animals may be in poor condition and still show the presence of good grooming. We suppose that the stress induced by the administration of the vehicle and the carcinogen will have the same effect on the animals grooming. As previously reported, animals exhibiting different self-grooming patterns may not show differences in behavioral levels related to stress factors [25]. Knowing this, grooming should be a parameter to be analyzed with more attention in these protocols, not only ensuring that the behavior is obvious, but also considering factors such as pattern, frequency, and intensity. The use on new strategies may also be explored. For example, grooming can best be assessed by applying a nontoxic fluorescent oil to the rodent and then measuring how fast the animal self-cleans [26].

Some induced animals exhibited a swollen abdomen, resulting in altered body conditions, which after being euthanized showed a pasty content accumulated in the caecum and the presence of gases in the remaining colon. The change in stool appearance, i.e., diarrhea, was expected, since the change in stool caliber is a characteristic symptom of CRC [27].

When planning the trial for the CRC, we did not find HE tables specific for the model, so we adapted those previously used in the animal models of mammary and urinary bladder cancers, hoping that they would also provide an indication of the real discomfort existing in the CRC model. Although these studies have not registered changes in the parameters evaluated in Table 1, our study showed that these parameters are more sensitive for this model of chemically induced CRC [5,9]. Some animals showed other changes not included in the HE table, namely BW increase and priapism. Despite being a parameter commonly evaluated in animal models of cancer to assess the severity of the disease, the animals’ BW has some limitations since it may be masked by the development of ascites or the increase of tumors mass [28]. In view of our results, monitoring the variation in the BWs of animals in this model of CRC is not a very trustworthy parameter, because intestinal impaction and ascites constitute a false weight gain. For that reason, the reported animals’ BWs were corrected to the weight of the intestinal tissue and its content. Although our results showed only a significant increase in the corrected BWs of group CTRL2 when compared to those of group CRC1, in a general way, the BWs of the control groups were higher than those of the induced groups. Our results are similar to those previously published by Jia and Han [29], who reported a decrease in the BWs of the DMH-exposed animals. This loss of BW may be suggestive of cachexia, a condition characteristic of cancer patients at advanced stages of the disease [11]. Variations in BW among animals from the same group can be explained by the rat strain used in this study. Indeed, we used an outbred strain, so there was heterogeneity in the BW variation of the animals that died until they showed a sudden increase of about 6–10% of their BWs. However, in our study, no animal exhibited such an advance neoplastic disease, and the decrease in BW might be associated with DMH proinflammatory effects. Priapism is a pathological condition characterized by a prolonged erection (about four hours or more), which is usually painful and is not due to any sexual stimulation [30,31]. Neurological and metabolic diseases, as well as the excessive use of drugs, have been associated with this condition [30]. To the best of our knowledge, this pathological condition has not been previously reported for this animal model of chemically induced CRC. We suppose that the distention of the abdomen may have some influence on the nerves that control the retractor muscle of the penis, leading to priapism.

BW increase and priapism appeared in the animals that died early with the same pathological pattern. Since some parameters were altered during our study and considering the number of precocious and unexpected deaths, we assume that this HE table can be improved to evaluate the animals’ condition by adding other new clinical signs not previously described (BW increase and priapism) and other relevant biological parameters to the guidelines. Eventually, individual housing could be recommended to monitor their evolution more closely with daily assessment. However, this situation of isolation can be an added factor of stress. Thus, animals housed in groups must be clearly marked and evaluated daily, anticipating the withdrawal of the protocol if necessary. The use of analgesia or opioid drugs may be useful in future studies to alleviate some of the more severe symptoms in this chemical induction model. However, it should be noted that both pain and the agents used to alleviate it have the potential to act as confounding factors in cancer research studies [32]. New information that pain relief, rather than being confounding, might both improve welfare and enhance study validity [33] are contradicted by more classical studies showing harmful adverse effects of analgesics use in cancer models. The use of opioids in animal models can bring other harmful adverse effects to them, such as hormonal changes and increase or decrease food intake, and can act through the sympathetic nervous system to cause hyperglycemia and decreased secretion of insulin [34].

Thermography is a noninvasive method to determine body temperature changes, inflammation, and tumors’ vascularization [9,35]. According to Banic et al. [36], the regions with higher temperature in thermographic images of DMH-exposed animals with the swollen abdomen corresponded to the tumor origin, i.e., the colon. Our data did not show changes in the temperature measured on the animals’ back by thermography, but we observed an increase in the abdominal temperatures of the animals belonging to group CRC2, when compared with those belonging to group CTRL2. Groups CTRL1 and CRC1 sacrificed at 11 weeks after DMH administration did not exhibit differences in surface abdominal temperature. It should be noted that thermographic imaging of surface temperature provides indirect measure of tumor vascularization, and this information can be complemented with other widely used imaging techniques, such as computed tomography, magnetic resonance imaging, position emission tomography, ultrasonography, and endoscopy. These in vivo techniques allow early detection and cancer staging [35,37,38,39]. We consider that researchers using this model should follow a renewed table of HEs in their research (Table S3, Supplementary Materials). This new table comprises information from this study and “chromodachryorrhea”, which has been suggested as an noninvasive method to assess disturbance and stress in animals [15].

## 5. Conclusions

Our results suggested that during CRC induction by DMH, Wistar rats showed a characteristic pathological pattern, exhibiting a sudden increase in BW, swollen abdomen, diarrhea, and priapism. Five animals died prematurely, before the previously determined score. Considering these results, the HE table must be improved. Thus, for future work we suggest the inclusion of the following parameters: thermography evaluation of abdomen, priapism, and BW gain. Furthermore, the observers should be blinded to the experimental groups. Considering the lack of published works concerning HEs in animal models of CRC, we believe that this work will help researchers to apply the 3Rs policy in the implementation of this protocol.

## Figures and Tables

**Table 1:** Used scoring sheet for colorectal carcinogenesis chemically induced by dimethylhydrazine (DMH), adapted from the Canadian Council on Animal Care (CCAC) guidelines [5,9].

	Parameter	Score			
		0	1	2	3
General appearance and state of consciousness of the animal	Body condition	Good	Altered body condition	Emaciated	---
	Body weigth (BW)	Normal	Loss of <10%	Loss of 10–20%	Loss of >20% (euthanasia)
	Posture	Normal posture	Posture changes (orthopnea posture)	---	---
	Hair appearance and grooming	Normal	Lack of grooming	Bad-looking hair and chromodachryorrhea	Chromodachryorrhea and hair with a very bad appearance
	Mucous color	Normal	Slightly anemic	Moderately anemic	Severe anemia
	Eyes, ears, and whiskers	Normal	Partially closed eyes, droopy ears, and forward whiskers	Completely closed eyes, droopy and curved ears, and forward and bunched whiskers	---
	Mental status	Normal	Lethargic	---	Stupor/coma (euthanasia)
Behavior	Response to external stimuli	Normal	Moderate response	Moderate response with vocalization	Violent response
Clinical signs	Hydration status	Normal	Abnormal skin pinch test (>2 s)	---	---
	Stool appearance	Normal	Diarrhea	Black (digested blood)	Bloody stool
	Convulsions	Absence	---	---	Presence
	Dimension of damage caused by injections	Without skin lesion	Lesion with diameter ≤8 mm	Lesion with diameters of ≥9 and ≤14 mm	Lesion with diameters of ≥15 mm
	Macroscopic appearance of induced skin lesions	Absence of necrosis	---	---	Presence of necrosis (euthanasia)
	Infection of induced skin lesions	Absence of infection	---	---	Presence of inflammatory exudate (euthanasia)

**Table 2:** Mean of the scores per group, considering the scores registered for each animal according to the sheet of HEs. Data are presented as mean ± SD.

Group	Mean ± SD
CTRL1	0.860 ± 0.283
CRC1	0.695 ± 0.517
CTRL2	0.593 ± 0.293
CRC2	1.021 ± 0.488 ^a^

^a^ Statistically different from group CTRL2 (*p* < 0.05). The control groups (CTRL1 and CTRL2) were injected with EDTA–saline solutions, and the induced groups are represented by CRC1 and CRC2. Groups 1 and 2 were euthanized 11 and 17 weeks after the first administration, respectively.

**Table 3:** Altered parameters in the animals from the induced groups (CRC1 and CRC2) that died due to hemorrhagic enteritis during the study.

Group	Animal	Cause of Death	Changed Parameter	Week
CRC1	1	Hemorrhagic enteritis	Approximately 10% increase in BW	5
			Swollen abdomen	6
			Diarrhea	6
			Priapism	8
			Death	10
CRC2	2		Approximately 10% increase in BW	7
			Swollen abdomen	8
			Diarrhea	10
			Priapism	12
			Death	14
	3		Approximately 6% increase in BW	9
			Swollen abdomen	10
			Diarrhea	11
			Priapism	14
			Death	16
	4		Swollen abdomen	15
			Diarrhea	16
			Death	17

**Table 4:** Ponderal gains (%) of animals before and after intraperitoneal administrations and one week before a sudden increase in BW.

Animal	Ponderal Gain (%)			
	*Acclimatization Period*	*1st Week of Administration*	*Week before the BW Increase*	*Week of a Sudden BW Increase*
1	15.60	7.61	−6.73	10.49
2	16.04	10.91	2.11	6.40
3	14.57	9.27	2.11	9.48

## Data Availability

Data is contained within the article or supplementary material.

## Supplementary Materials

The following are available online at https://www.mdpi.com/article/10.3390/ani11040985/s1, Table S1: Initial and final corrected BWs and food and water intake in the first and last weeks of the experimental protocol. All data are presented as mean ± SD, Figure S1. Animals’ mean body weight per week. Control groups (CTRL1 and 2) injected with ethylenediamine tetraacetic acid (EDTA)-saline and induced groups (CRC1 and 2) injected with 1,2-dimethylhydrazine (DMH). Groups 1 and 2 were euthanized 11 and 17 weeks after the first administration, respectively, Figure S2. Animals’ mean back and abdominal body temperature per week. Control groups (CTRL1 and 2) injected with ethylenediamine tetraacetic acid (EDTA)-saline and induced groups (CRC1 and 2) injected with 1,2-dimethylhydrazine (DMH). Groups 1 and 2 were euthanized 11 and 17 weeks after the first administration, respectively, Table S2: Individual score assigned to each animal per week, Table S3: New scoring sheet for colorectal carcinogenesis chemically induced by DMH. Adapted from the CCAC guidelines, Mason et al., Oliveira et al., and Faustino-Rocha et al. Parameters added after this work are demarked in bold.
