# Supplementary material for: Refinement of Animal Model of Colorectal Carcinogenesis through the Definition of Novel Humane Endpoints
Source: Animals (Basel). 2021 Apr 1;11(4):985. doi: 10.3390/ani11040985 (PMC8066901; doi:10.3390/ani11040985)
Supplement: Supplementary file 1 [file animals-11-00985-s001.pdf]

## Supplementary Materials

*Article*

# Refinement of animal model of colorectal carcinogenesis through the definition of novel humane endpoints

**Rita Silva-Reis[1], Ana I. Faustino-Rocha[1,2], Mariana Gonçalves[1], Catarina Castro Ribeiro[1], Tiago Ferreira[1], CM Ribeiro-Silva[1], Lio Gonçalves[3,4], Luís Antunes[1,5], Carlos Venâncio[1,6], Rita Ferreira[7], Adelina Gama[5,8], Paula A. Oliveira[1,7]**

[1] Center for the Research and Technology of Agro-Environmental and Biological Sciences (CITAB), University of Trás-os-Montes and Alto Douro (UTAD), Vila Real, Portugal
[2] Department of Zootechnics, School of Sciences and Technology, University of Évora, Évora, Portugal
[3] Engineering Department, UTAD, Vila Real, Portugal
[4] Institute for Systems and Computer Engineering, Technology and Science, Porto, Portugal
[5] Department of Veterinary Sciences, UTAD, Vila Real, Portugal
[6] Department of Animal Sciences, UTAD, Vila Real, Portugal
[7] Associated Laboratory for Green Chemistry (REQUIMTE), Department of Chemistry, University of Aveiro (UA), Aveiro, Portugal
[8] Animal and Veterinary Research Center (CECAV), UTAD, Vila Real, Portugal
* Correspondence: pamo@utad.pt

Received: date; Accepted: date; Published: date

**Table S1.** Initial and final corrected BW, food and water intake in the first and last weeks of the experimental protocol. All data is presented as mean ± S.D.

| Group | Body weight (g) | | Food consumption (g) | | Water consumption (ml) | |
|---|---|---|---|---|---|---|
| | Initial | Final | Initial | Final | Initial | Final |
| CTRL1 (n=6) | 223.4 ± 10.2 | 409.0 ± 19.7 | 20.7 ± 0.2 | 26.3 ± 0.2 | 29.7 ± 2.6 | 26.3 ± 0.7 |
| CRC1 (n=8) | 222.4 ± 4.3 | 366.4 ± 38.9 [a] | 23.2 ± 0.7 | 25.6 ± 1.7 | 25.9 ± 4.5 | 20.9 ± 3.8 |
| CTRL2 (n=6) | 229.1 ± 14.6 | 441.1 ± 34.8 | 23.7 ± 0.1 | 27.3 ± 1.1 | 29.6 ± 0.5 | 23.0 ± 0.7 |
| CRC2 (n=9) | 225.8 ± 7.9 | 393.9 ± 36.7 | 23.8 ± 0.4 | 27.8 ± 0.6 | 28.1 ± 0.9 | 20.9 ± 1.9 |

[a] Statistically different from group CTRL2 ($p$<0.05). Control groups (CTRL1 and 2) injected with EDTA-saline and induced groups (CRC1 and 2). Groups 1 and 2 were euthanized 11 and 17 weeks after the first administration, respectively.

**Figure S1.** Animals' mean body weight *per* week. Control groups (CTRL1 and 2) injected with ethylenediamine tetraacetic acid (EDTA)-saline and induced groups (CRC1 and 2) injected with 1,2-dimethylhydrazine (DMH). Groups 1 and 2 were euthanized 11 and 17 weeks after the first administration, respectively.

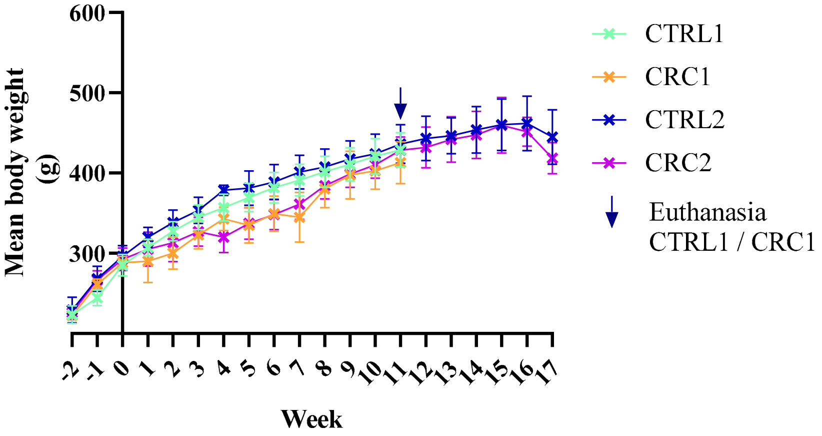


**Figure S2.** Animals' mean back and abdominal body temperature *per* week. Control groups (CTRL1 and 2) injected with ethylenediamine tetraacetic acid (EDTA)-saline and induced groups (CRC1 and 2) injected with 1,2-dimethylhydrazine (DMH). Groups 1 and 2 were euthanized 11 and 17 weeks after the first administration, respectively.

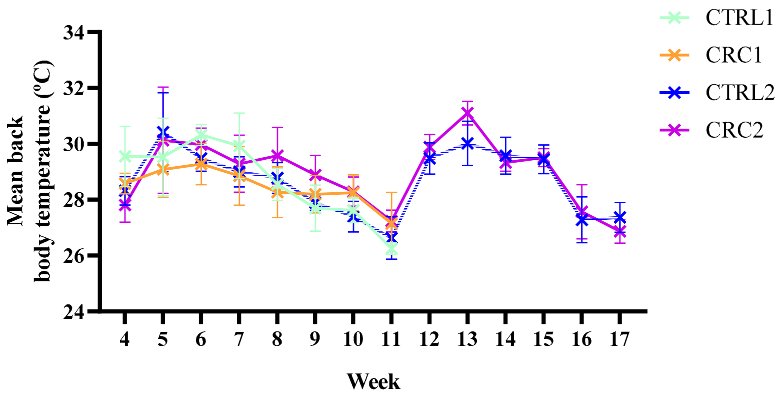


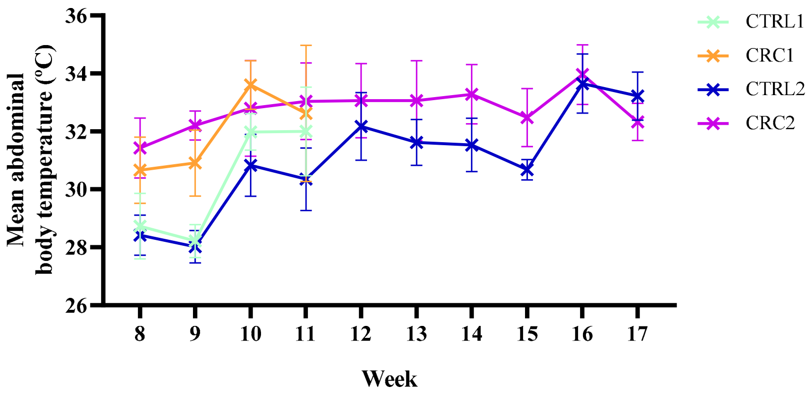


cor

**Table S1**. Individual score assigned to each animal *per* week.

| Group | Rat | Individual HE score per week | | | | | | | | | | | | | | | | |
|---|---|---|---|---|---|---|---|---|---|---|---|---|---|---|---|---|---|---|
| | | **1** | **2** | **3** | **4** | **5** | **6** | **7** | **8** | **9** | **10** | **11** | **12** | **13** | **14** | **15** | **16** | **17** |
| CTRL1 | 1 | 1[d] | 1[d] | 1[d] | 1[d] | 1[d] | 1[d] | 1[d] | 1[d] | 1[d] | 1[d] | 1[d] | | | | | | |
| | 2 | 1[d] | 1[d] | 1[d] | 1[d] | 1[d] | 1[d] | 1[d] | 1[d] | 1[d] | 1[d] | 1[d] | | | | | | |
| | 3 | 0 | 1[d] | 1[d] | 1[d] | 1[d] | 1[d] | 1[d] | 1[d] | 1[d] | 1[d] | 1[d] | | | | | | |
| | 4 | 1[d] | 1[d] | 0 | 0 | 0 | 1[d] | 1[d] | 1[d] | 1[d] | 1[d] | 1[d] | | | | | | |
| | 5 | 1[d] | 1[d] | 1[d] | 1[d] | 1[d] | 1[d] | 1[d] | 1[d] | 1[d] | 1[d] | 1[d] | | | | | | |
| | 6 | 0 | 0 | 0 | 0 | 0 | 0 | 0 | 0 | 0 | 1[d] | 1[d] | | | | | | |
| CRC1 | 1 | 0 | 0 | 0 | 0 | 0 | 1[g] | 2[b, g] | | | | | | | | | | |
| | 2 | 0 | 0 | 0 | 0 | 0 | 0 | 0 | 1[d] | 1[d] | 1[d] | 1[d] | | | | | | |
| | 3 | 0 | 0 | 0 | 0 | 0 | 2[a,j] | 2[a,j] | 2[a,j] | 3[a,d,j] | | | | | | | | |
| | 4 | 3[b,d,c] | 0 | 0 | 1[d] | 1[d] | 1[d] | 1[d] | 1[d] | 1[d] | 1[d] | 1[d] | | | | | | |
| | 5 | 0 | 0 | 0 | 0 | 0 | 0 | 1[d] | 1[d] | 1[g] | 2[a,g] | 1[a] | | | | | | |
| | 6 | 0 | 1[d] | 0 | 1[d] | 1[d] | 1[d] | 0 | 0 | 0 | 0 | 0 | | | | | | |
| | 7 | 0 | 0 | 0 | 0 | 0 | 0 | 0 | 0 | 0 | 1[d] | 0 | | | | | | |
| | 8 | 0 | 1[d] | 1[d] | 1[d] | 0 | 0 | 1[d] | 1[d] | 1[d] | 2[a,d] | 2[a,d] | | | | | | |
| CTRL2 | 1 | 1[d] | 1[d] | 1[d] | 1[d] | 1[d] | 1[d] | 1[d] | 1[d] | 1[d] | 1[d] | 1[d] | 1[d] | 1[d] | 1[d] | 1[d] | 1[d] | 1[d] |
| | 2 | 0 | 0 | 0 | 0 | 1[d] | 1[d] | 1[d] | 1[d] | 1[d] | 1[d] | 1[d] | 1[d] | 1[d] | 1[d] | 1[d] | 1[d] | 1[d] |
| | 3 | 0 | 1[d] | 1[d] | 1[d] | 1[d] | 1[d] | 1[d] | 1[d] | 1[d] | 1[d] | 1[d] | 1[d] | 1[d] | 1[d] | 1[d] | 1[d] | 1[d] |
| | 4 | 0 | 0 | 0 | 0 | 0 | 0 | 0 | 0 | 0 | 0 | 0 | 0 | 0 | 0 | 0 | 0 | 0 |
| | 5 | 0 | 0 | 0 | 1[d] | 0 | 0 | 0 | 0 | 0 | 0 | 0 | 0 | 1[d] | 1[d] | 1[d] | 1[d] | 1[d] |
| | 6 | 0 | 0 | 0 | 0 | 0 | 0 | 0 | 0 | 0 | 1[d] | 1[d] | 1[d] | 1[d] | 1[d] | 1[d] | 1[d] | 1[d] |
| CRC2 | 1 | 0 | 0 | 0 | 0 | 0 | 0 | 0 | 0 | 1[d] | 2[a,d] | 3[a,d,j] | 3[a,d,j] | 3[a,d,j] | 3[a,d,j] | 3[a,d,j] | | |
| | 2 | 1[d] | 0 | 0 | 1[d] | 1[d] | 1[d] | 1[d] | 1[d] | 1[d] | 1[d] | 2[d,g] | 1[d] | 1[d] | 1[d] | 2[a,d] | 3[a,d,j] | |
| | 3 | 1[d] | 0 | 3[l] | 3[l] | 0 | 0 | 2[d,j] | 2[d,j] | 2[d,j] | 2[d,g] | 2[d,g] | 0 | 0 | 0 | 1[d] | 1[d] | 1[d] |
| | 4 | 0 | 0 | 0 | 0 | 0 | 0 | 0 | 1[d] | 1[d] | 1[d] | 1[d] | 1[d] | 1[d] | 1[d] | 2[d,g] | 2[d,g] | 2[d,g] |
| | 5 | 0 | 1[d] | 1[d] | 0 | 0 | 0 | 0 | 0 | 0 | 0 | 2[d,g] | 2[d,g] | 2[d,g] | 1[d] | 1[d] | 1[d] | 1[d] |
| | 6 | 0 | 0 | 0 | 1[d] | 1[d] | 2[d,g] | 0 | 0 | 1[d] | 1[d] | 2[d,g] | 1[d] | 0 | 1[d] | 1[d] | 1[d] | 1[d] |
| | 7 | 0 | 0 | 0 | 1[d] | 1[d] | 2[d,g] | 0 | 1[d] | 1[d] | 1[d] | 1[d] | 1[d] | 1[d] | 1[d] | 1[d] | 1[d] | 2[a,d,j] |
| | 8 | 0 | 1[d] | 1[d] | 1[d] | 1[d] | 2[a,d] | 2[a,d] | 1[a] | 2[a,d] | 2[a,d] | 2[a,d] | 2[a,d] | 2[a,d] | 2[a,d] | 3[a,d,j] | 3[a,d,j] | 3[a,d,j] |
| | 9 | 1[d] | 1[g] | 1[d] | 1[d] | 2[d,g] | 1[d] | 1[d] | 2[a,d] | 2[a,d] | 3[a,d,j] | 3[a,d,j] | 3[a,d,j] | 3[a,d,j] | | | | |

[a] Body condition; [b] Body weight; [c] Posture; [d] Hair appearance and grooming; [e] Mucous Color; [f] Eyes, ears and whiskers; [g] Mental Status; [h] Response to external stimuli; [i] Hydration status; [j] Stool appearance; [k] Convulsions; [l] Dimension of damage caused by injections; [m] Macroscopic appearance of induced skin lesions; [n] Infection of induced skin lesions. Control groups (CTRL1 and 2) injected with ethylenediamine tetraacetic acid (EDTA)-saline and induced groups (CRC1 and 2) injected with 1,2-dimethylhydrazine (DMH). Groups 1 and 2 were euthanized 11 and 17 weeks after the first administration, respectively.

**Table S3.** New Scoring sheet for colorectal carcinogenesis chemically-induced by 1,2-dimethylhydrazine (DMH). Adapted from CCAC guidelines, Mason et al. (2004), Oliveira et al. (2017), Faustino-Rocha et al. (2019) [1–3]. Parameters added after this work are demarked in bold.

| | Parameter | Score 0 | Score 1 | Score 2 | Score 3 |
|---|---|---|---|---|---|
| General appearance and state of consciousness of the animal | **Body condition** | Good | **Underconditioned** | Emaciated | **Swollen abdomen and priapism** |
| | **Body mass*** | Normal | Loss/**Gain 5-10%** | Loss/**Gain** 10-20% | Loss/**Gain** >20% |
| | Posture | Normal posture | Posture changes (**hunched** posture) | --- | --- |
| | Aspect of hair and grooming | Normal | Lack of grooming | **---** | **---** |
| | **Chromodachryorrhea** | **Absence** | **Slight** | **Noticeable** | **Well-marked** |
| | **Abdominal Temperature** | **To be determined in a future work.** | | | |
| | Mucous color | Normal | Slightly anemic | Moderately anemic | Severe anemia |
| | **Grimace changes** | Normal | **---** | **Partially closed eyes, droopy ears, forward whiskers** | **Completely closed eyes, droopy and curved ears, forward and bunched whiskers** |
| | Mental status | Normal | **Slight apathy** | **Inactive** | Stupor / Coma |
| Behavior | Response to external stimuli | Normal | Moderate response | Moderate response with vocalization | Violent response |
| Clinical signs | Hydration status | Normal | --- | **Abnormal skin pinch test (> 2 sec.)** | --- |
| | Appearance of stool | Normal | Diarrhea | Black (digested blood) | Bloody Stool |

| | | | | |
|---|---|---|---|---|
| Convulsions | Absence | --- | --- | Presence |
| Dimension of damage caused by injections | Absence | Lesion with diameter ≤8mm | Lesion with diameter≥ 9 and ≤14 mm | Lesion with diameter≥ 15 mm |
| Macroscopic appearance of induced skin lesions | Absence | --- | --- | Presence of necrosis |
| Infection of induced skin lesions | Absence | --- | --- | Presence of inflammatory exudate |

* The body weight parameter must consider the body weight increase due to growth, being adjusted for each study

**Recommendation - A total clinical score of 6 or a score of 3 in a single parameter indicates the need of urgent euthanasia.**

## References


1. Mason, G.; Wilson, D.; Hampton, C.; Würbel, H. Non-invasively Assessing Disturbance and Stress in Laboratory Rats by Scoring Chromodacryorrhoea. *Altern. to Lab. Anim.* **2004**, *32*, 153–159, doi:10.1177/026119290403201s25.
2. Faustino-Rocha, A.I.; Ginja, M.; Ferreira, R.; Oliveira, P.A. Studying humane endpoints in a rat model of mammary carcinogenesis. *Iran. J. Basic Med. Sci.* **2019**, *22*, 643–649, doi:10.22038/ijbms.2019.33331.7957.
3. Oliveira, M.; Nascimento-gonçalves, E.; Silva, J.; Oliveira, P.A.; Ferreira, R.; Antunes, L.; Arantes-rodrigues, R.; Faustino-rocha, A.N.A.I. Implementation of Human Endpoints in a Urinary Bladder Carcinogenesis Study in Rats. *In Vivo (Brooklyn).* **2017**, *31*, 1073–1080, doi:10.21873/invivo.11172.
